# G‐CSF induces CD15^+^ CD14^+^ cells from granulocytes early in the physiological environment of pregnancy and the cancer immunosuppressive microenvironment

**DOI:** 10.1002/cti2.1395

**Published:** 2022-05-17

**Authors:** Ebtehag Maneta, Livingstone Fultang, Jemma Taylor, Matthew Pugh, William Jenkinson, Graham Anderson, Arri Coomarasamy, Mark D Kilby, David M Lissauer, Francis Mussai, Carmela De Santo

**Affiliations:** ^1^ 1724 Institute of Immunology and Immunotherapy University of Birmingham Birmingham UK; ^2^ 1724 Tommy’s National Centre for Miscarriage Research Institute of Metabolism and Systems Research University of Birmingham Birmingham UK; ^3^ 1724 Institute of Metabolism and Systems Research University of Birmingham Birmingham UK; ^4^ Fetal Medicine Centre Birmingham Women’s & Children’s Foundation Trust Edgbaston, Birmingham UK; ^5^ Institute of Life Course and Medical Sciences University of Liverpool Liverpool UK; ^6^ Malawi‐Liverpool‐Wellcome Trust Blantyre Malawi

**Keywords:** cancer, CD15^+^CD14^+^, G‐CSF, MDSC, placenta

## Abstract

**Objectives:**

Recombinant granulocyte colony‐stimulating factor (G‐CSF) is frequently administered to patients with cancer to enhance granulocyte recovery post‐chemotherapy. Clinical trials have also used G‐CSF to modulate myeloid cell function in pregnancy and inflammatory diseases. Although the contribution of G‐CSF to expanding normal granulocytes is well known, the effect of this cytokine on the phenotype and function of immunosuppressive granulocytic cells remains unclear. Here, we investigate the impact of physiological and iatrogenic G‐CSF on an as yet undescribed granulocyte phenotype and ensuing outcome on T cells in the settings of cancer and pregnancy.

**Methods:**

Granulocytes from patients treated with recombinant G‐CSF, patients with late‐stage cancer and women enrolled on a trial of recombinant G‐CSF were phenotyped by flow cytometry. The ability and mechanism of polarised granulocytes to suppress T‐cell proliferation were assessed by cell proliferation assays, flow cytometry and ELISA.

**Results:**

We observed that G‐CSF leads to a significant upregulation of CD14 expression on CD15^+^ granulocytes. These CD15^+^CD14^+^ cells are identified in the blood of patients with patients undergoing neutrophil mobilisation with recombinant G‐CSF, and physiologically in women early in pregnancy or in those treated as a part of a clinical trial. Immunohistochemistry of tumor tissue or placental tissue identified the expression of G‐CSF. The G‐CSF upregulates the release of reactive oxygen species (ROS) in CD15^+^CD14^+^ cells leading to the suppression of T‐cell proliferation.

**Conclusions:**

G‐CSF induces a population of ROS^+^ immunosuppressive CD15^+^CD14^+^ granulocytes. Strategies for how recombinant G‐CSF can be scheduled to reduce effects on T‐cell therapies should be developed in future clinical studies.

## Graphical abstract

G‐CSF physiologically drives granulocyte expansion and is also administered in recombinant form alongside chemotherapy or immunotherapy. We show that G‐CSF upregulates the release of reactive oxygen species (ROS) in a unique population of CD15^+^CD14^+^ cells leading to the suppression of T‐cell proliferation.

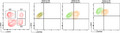

## Introduction

Myeloid‐derived suppressor cells (MDSCs) are a heterogeneous population of myeloid cells that play a critical role in maintaining immune tolerance.^1^ In humans, MDSCs are defined as CD33^+^CD11b^+^ cells and further classified into two distinct subsets: monocytic (M‐MDSC) identified as CD14^+^ HLA‐DR^low/−^ CD15^−^ cells, and granulocytic (G‐MDSC) characterised by CD15^+^ HLA‐DR^low/−^ CD14^−^ collected from the peripheral blood mononuclear cell (PBMC) layer after density centrifugation, a crude but critical step in MDSC isolation.^2^ We and others have shown that MDSCs may functionally suppress T‐cell proliferation by the upregulation of immunosuppressive pathways such as arginine or cysteine depletion, inducible nitric oxide synthase (iNOS) or NADPH oxidase expression, or by the release of cytokines such as IL‐10.^3^, ^4^ Cancer cells may directly drive MDSC expansions through the release of cytokines such as TGF‐β and GM‐CSF, leading to escape from immunosurveillance.^5^, ^6^

The physiological expansion of MDSC populations to modulate potentially damaging inflammatory responses in states such as pregnancy or early life is also increasingly recognised.^7^, ^8^ Several murine studies identify that MDSCs play a key early role in healthy pregnancies. Peripheral blood MDSC numbers rise within days of fertilisation and support implantation of the embryo. The absence of G‐MDSCs leads to miscarriage, and by enhancing MDSC activity early in pregnancy, miscarriages can be reduced in murine models.^9^ In human pregnancy, MDSCs are substantially increased in the peripheral blood and placentae of healthy pregnant women during all stages of pregnancy with a less pronounced rise in women with a history of recurrent miscarriage.^10^ The factors that induce MDSCs and drive their suppressive activity in human pregnancy remain an ongoing area of research.

In contrast to these endogenous responses, recombinant cytokines notably G‐CSF have long‐standing use in chemotherapy regimens to shorten the duration and risks of myelosuppression.^11^ Myeloid‐inducing recombinant cytokines may be further used alongside immunotherapies as adjuncts to boost autologous and engineered immunity.^12^, ^13^ Outside of cancer, recombinant G‐CSF has been used to modulate the immune microenvironment by inducing neutrophil expansion to support antimicrobial activity, or reverse inflammation through myeloid‐derived suppressor cell (MDSC) induction.^14^, ^15^ We have recently reported such an approach in the context of a Phase II clinical trial using recombinant G‐CSF to reduce the risk of miscarriage.^16^

However, physicians should be cautious in how cytokines are used, as myeloid cells create a number of negative influences in the tumor microenvironment including promotion of tumor progression and suppression of CAR‐T activity.^17^ Although peripheral blood granulocytes may be thought of as terminally differentiated, evidence from studies of cancer and inflammation indicates granulocytes have considerable plasticity.^18^ Such findings have led to renewed attempts to explore administering intensive multidrug induction chemotherapy (COJEC), without the usual use of G‐CSF.^19^ Here, we investigate the impact of physiological and iatrogenic rise in G‐CSF concentrations on an as yet undescribed granulocyte phenotype and ensuing outcome on T cells in the settings of cancer and pregnancy.

## Results and discussion

### G‐CSF polarises peripheral blood granulocytes to a CD14^+^ immunosuppressive phenotype (GM‐MDSC)

**Figure 1:**
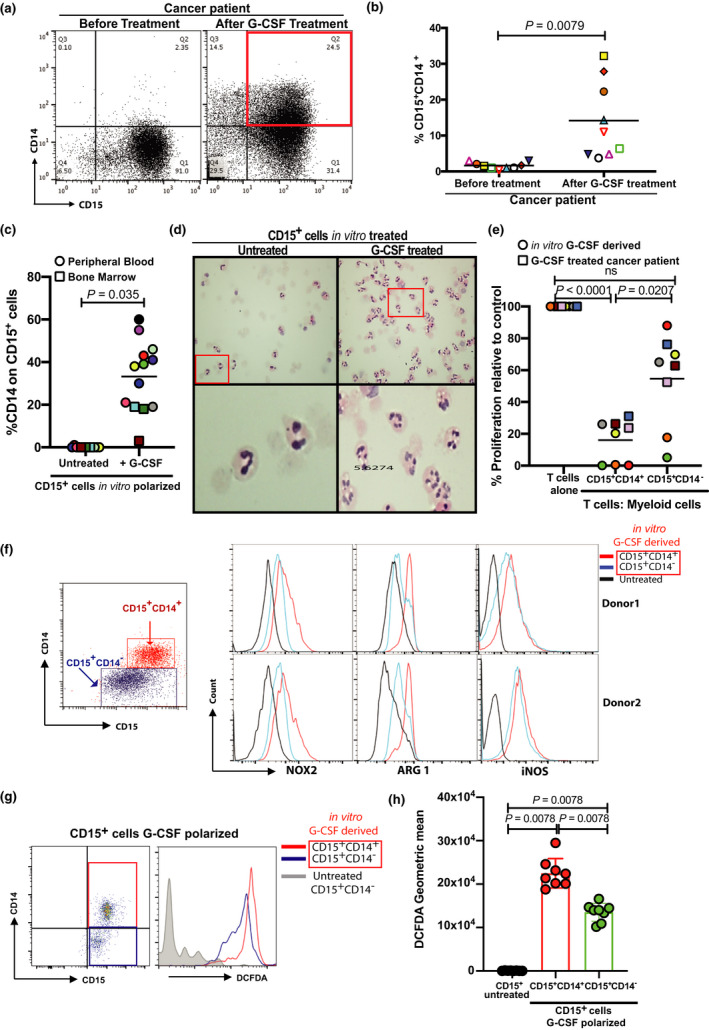
CD15^+^CD14^+^ cells are expanded post‐recombinant G‐CSF and suppress T‐cell responses. Frequency of CD15^+^CD14^+^ cells in the whole blood of cancer patients treated with recombinant G‐CSF. **(a)** Representative flow cytometry gating and **(b)** pooled analysis of *n* = 9 patients. **(c)** Frequency of CD15^+^CD14^+^ cells following treatment of peripheral blood granulocytes or whole bone marrow from healthy donors, with G‐CSF *ex vivo* for 48 h. **(d)** Haematoxylin and eosin staining of CD15^+^CD14^+^ cells demonstrating multilobed granulocytes, following healthy donor granulocytes treated with G‐CSF *in vitro* (upper = 10× and lower = red box 40×). **(e)** T‐cell proliferation in the presence of *in vitro* G‐CSF‐derived paired CD15^+^CD14^+^ or CD15^+^CD14^−^ cells or cells isolated directly from cancer patients treated with G‐CSF (ratio: 1 T cell: 0.5 myeloid), stimulated by anti‐CD3/CD28 antibodies for 96 h as measured by 3H‐thymidine incorporation. **(f)** Representative flow cytometry gating of CD15^+^CD14^−^ untreated controls or *in vitro* G‐CSF‐derived paired CD15^+^CD14^+^ or CD15^+^CD14^−^ cells from *n* = 2 donors, showing intracellular staining or NOX2, ARG1 or iNOS expression. **(g)** Representative flow cytometry gating of CD15^+^CD14^−^‐untreated controls or *in vitro* G‐CSF‐derived paired CD15^+^CD14^+^ or CD15^+^CD14^−^ cells from *n* = 2 donors, showing intracellular DCFDA staining corresponding to ROS production and **(h)** pooled analysis of *n* = 8 experiments.

Recombinant G‐CSF is regularly administered to patients with cancer to reduce the risk of neutropenic sepsis. Blood was sampled whilst patients with cancer were receiving daily subcutaneous recombinant G‐CSF post‐chemotherapy. Patients exhibited the expected expansion of total CD15^+^ cells; however, flow cytometry identifies a significant subpopulation of CD15^+^CD14^+^ cells in the peripheral blood mononuclear ring (Figure 1a and b, Supplementary figure 1a). Classical granulocytes are established as CD15^+^ but CD14^−^. We hypothesised the CD15^+^CD14^+^ cells could originate from bone marrow progenitors.^20^ We observed G‐CSF treatment led to an increased frequency of CD15^+^CD14^+^ cells from both healthy donor bone marrow cells and peripheral blood granulocytes (Figure 1c, Supplementary figure 1b and c). By contrast, CD14^+^ monocytes treated with G‐CSF did not increase CD15 expression (Supplementary figure 1d and e). An expansion of low‐density granulocytes compared with healthy donors is one of the characteristics of myeloid‐derived suppressor cells.^2^ Morphologically, CD15^+^CD14^+^ cells exhibited segmented nuclei consistent with a granulocytic lineage (Figure 1d and Supplementary figure 2a). CD15^+^CD14^+^ cells showed high expression of CD62L and CD16 compared with CD15^+^CD14^−^ cells (Supplementary figure 2b–g). Double‐positive cells were flow‐sorted either indirectly from *in vitro*‐derived G‐CSF‐treated CD15^+^ cells or directly from the blood of G‐CSF‐treated patients and placed in culture with allogeneic T cells where they suppressed the T‐cell proliferation (Figure 1e). A significant difference in suppressive potential was seen compared with CD15^+^CD14^−^ cells. Suppressive mediators were characterised by revealing an increased expression of NOX2, with minimal changes in ARG1 or iNOS expression (Figure 1f). Reactive oxygen species (ROS) production was higher in CD15^+^CD14^+^ cells than that in CD15^+^CD14^−^ cells, when both are derived from healthy donor granulocytes treated with G‐CSF (Figure 1g and h). No significant difference in intracellular cytokine profiles was seen after G‐CSF polarisation (Supplementary figure 3a).

### Expansion of CD15^+^CD14^+^ in cancer and pregnancy

To validate the presence of these cells in another cohort, we examined the peripheral blood of patients with cancer at diagnosis, identifying expansions of CD15^+^CD14^+^ circulating cells (Supplementary figure 3b). Although it is well established in murine models and patients with advanced cancer that MDSCs can be divided into monocytic (M‐MDSC) or granulocytic (G‐MDSC) phenotypes, one of the remaining challenges is to determine the temporal emergence of these cells and their phenotype. Notably in human studies, the majority of studies report patients with advanced cancer. By contrast, it is possible to follow the emergent immune response to an allogeneic organism, in the setting of pregnancy.

**Figure 2:**
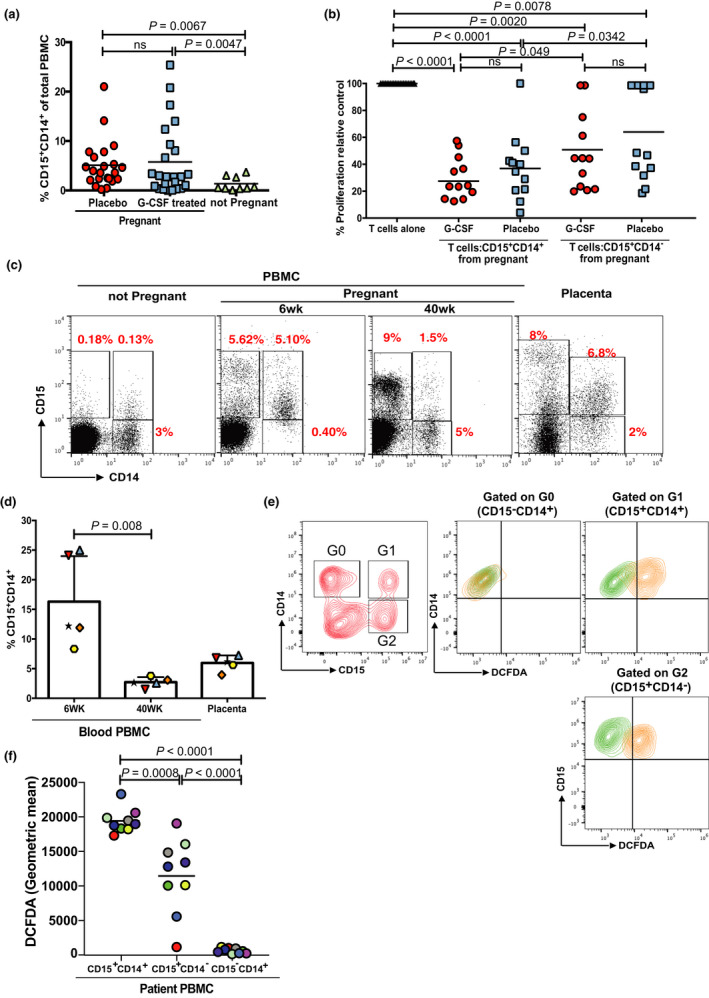
Frequency of myeloid cell populations in the PBMCs of pregnant women randomised to receive recombinant G‐CSF (*n* = 24) or placebo (*n* = 24) in the RESPONSE trial, and healthy non‐pregnant controls (*n* = 9). **(a)** Percentage of CD15^+^CD14^+^ in the PBMC layer of each patient group as measured by flow cytometry. **(b)** Addition of CD15^+^CD14^+^ cells or CD15^+^CD14^−^ cells (ratio: 1 T cell : 0.5 myeloid) from each patient group leads to the suppression of T‐cell proliferation as measured by ^3^H‐thymidine incorporation after 96 h. **(c)** Representative gating strategy and pooled analysis (*n* = 5) **(d)** of the frequency of CD15^+^CD14^+^ cells in the PBMCs of pregnant women at Week 6 and Week 40 and in decidual tissue at term, measured by flow cytometry. **(e)** Representative flow cytometry gating of DCFDA staining, indicating reactive oxygen species production, in populations of paired CD15^+^CD14^+^ or CD15^+^CD14^−^ from the blood of pregnant women in the RESPONSE trial and **(f)** pooled analysis (*n* = 9).

Maternal immunity plays a significant role in the maintenance or rejection of the allogeneic foetus and the successful outcome of pregnancy. In cases where pregnancy fails, T cells may contribute to the rejection of the allogeneic foetus.^21^ Indeed, we have shown that foetal‐specific CD8^+^ T cytotoxic T‐cell responses are detectable in the blood of pregnant women from the first trimester and that women with recurrent miscarriage have a peripheral immunological shift evidenced by an increase in Th1 and Th17 cells.^22^ Phenotyping of peripheral blood samples obtained at 6 weeks of gestation revealed no significant difference in the frequency of T cells in the blood of pregnant women compared with that of healthy non‐pregnant individuals (*P* = 0.09; Supplementary figure 3c). However, T cells had a significant reduction in proliferative potential (*P* = 0.0001; Supplementary figure 3d) suggesting the presence of a systemic immunosuppressive microenvironment. An evaluation of the blood of pregnant women randomised to receive recombinant G‐CSF (*n* = 45, at 6 weeks’ gestation) identified significant expansions in CD11b^+^CD15^+^CD14^−^ cells, and no difference in CD11b^+^CD15^−^CD14^+^ cells (Supplementary figure 4a and b). Notably, at this early time point a third population of CD11b^+^CD15^+^CD14^+^ cells are revealed in pregnant women treated with and without G‐CSF (Figure 2a), which express CD33 and HLA‐DR, consistent with our findings above (Supplementary figure 4c). Double‐positive cells sorted from peripheral blood of pregnant women and placed in culture with allogeneic T cells suppressed the T‐cell proliferation (Figure 2b). These CD14^+^CD15^+^ cells decreased by Week 40 and return towards background frequencies at parturition (Figure 2c and d).

### CD15^+^CD14^+^ cells impair T‐cell expansion via reactive oxygen species production

**Figure 3:**
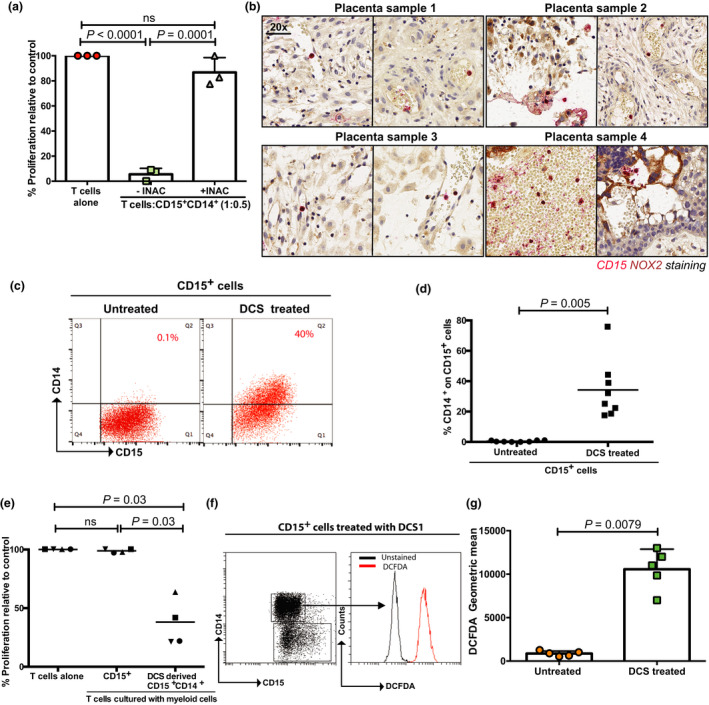
Decidual cells upregulate CD14 on CD15^+^CD14^−^ cells. **(a)** Addition of CD15^+^CD14^+^ cells (ratio: 1 T cell: 0.5 myeloid) isolated from the blood of the pregnant woman group leads to the suppression of T‐cell proliferation, which is rescued by the addition of iNAC, as measured by ^3^H‐thymidine incorporation after 96 h. **(b)** Immunohistochemistry of human first‐trimester placental tissue (*n* = 8) confirmed the presence of NOX2‐positive (red) CD15^+^‐infiltrating myeloid cells (brown). Representative staining from 4 placentas shown. **(c)** Culture of granulocytes with decidua‐conditioned supernatants or R10% (untreated) for 48 h leads to an upregulation of CD14 expression as demonstrated by representative flow cytometry gating with **(d)** pooled analysis (*n* = 8) **(e)** CD15^+^CD14^+^ cells, generated by culture of granulocytes in decidua‐conditioned media, suppress T‐cell proliferation (ratio: 1 T cell: 0.5 myeloid), stimulated by anti‐CD3/CD28 antibodies for 96 h as measured by ^3^H‐thymidine incorporation (*n* = 4). **(f)** DCFDA staining, indicating reactive oxygen species production, in populations of CD14^+^CD15^+^ cells generated from culture of granulocytes in decidua‐conditioned media for 48 h – representative flow cytometry gating and **(g)** pooled analysis (*n* = 5).

The haemochorial placenta is the major interface between the mother and the foetus in human pregnancy. An examination of the human placental tissue identified the presence of infiltrating CD15^+^CD14^+^ cells (Figure 2c and d). MDSCs may inhibit T‐cell proliferation through several different mechanisms. Analysis of patient‐derived CD15^+^CD14^+^ cells revealed an increased reactive oxygen species production, compared with CD15^+^CD14^−^ and CD15^−^CD14^+^ cells (Figure 2e and f). Treatment of CD15^+^CD14^+^ cells with the free radical scavenger N‐acetylcysteine (iNAC) rescued the T‐cell proliferation, confirming the role of ROS as the suppressive mediator (Figure 3a). Only minimal expression of the other classical suppressive mediators ARG1, iNOS, IL‐10 or IL‐13 was seen (Supplementary figure 5a). Supporting these findings, no significant changes in plasma arginine concentrations were seen (Supplementary figure 5b). Plasma IL‐10 and IL‐13 concentrations were low (Supplementary figure 5c). Immunohistochemistry of human placental tissue confirmed the presence of NOX2‐positive CD15^+^‐infiltrating myeloid cells (Figure 3b).

**Figure 4:**
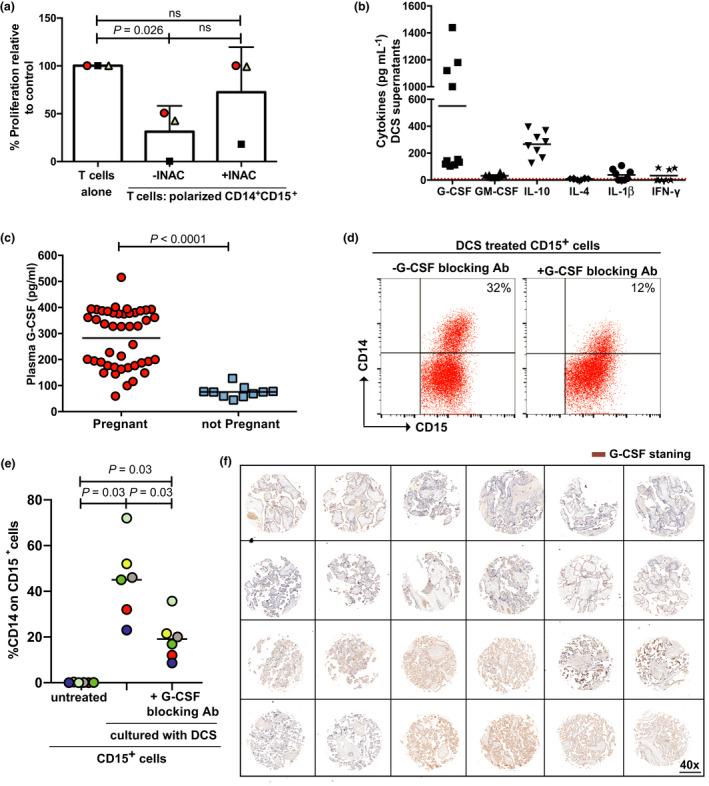
**(a)** Addition of iNAC, in the presence of decidua‐conditioned medium‐generated CD15^+^CD14^+^ (ratio: 1 T cell : 0.5 myeloid), leads to the rescue of T‐cell proliferation as measured by ^3^H‐thymidine incorporation after 96 h. **(b)** Cytokine ELISA of decidual cell supernatants (*n* = 10 donors; R10% = 0 ng L^−1^) and **(c)** patient plasma (*n* = 44 pregnant women and *n* = 10 non‐pregnant controls). **(d)** Addition of G‐CSF‐neutralising antibody to cultures of granulocytes in decidua‐conditioned media (48 h) reduces CD14 upregulation, as shown by representative flow cytometry staining and (e) pooled analysis (*n* = 6). **(f)** Representative immunohistochemistry of human placental tissue microarray demonstrating G‐CSF staining (brown) in the placentas of women at term (*n* = 12).

We hypothesised that the placenta (or maternal decidua) could release factors into the local microenvironment to induce CD15^+^CD14^+^ development. The culture of healthy donor granulocytes with the supernatant of sorted decidual cells from human placentas led to an upregulation of CD14 on CD15^+^ granulocytes (Figure 3c and d), which suppressed the T‐cell proliferation (Figure 3e and Supplementary figure 6a). Decidual supernatants upregulated ROS production in CD15^+^CD14^+^ cells (Figure 3f and g), which on inhibition rescued the T‐cell proliferation (Figure 4a). Granulocytes from the spleens of naïve mice conditioned with murine placental supernatants (G‐CSF positive; Supplementary figure 6b) showed an increased expression of Ly6C (Supplementary figure 6c) and similarly suppress T‐cell proliferation (Supplementary figure 6d).

ELISA of decidual cell supernatants and patient plasma confirmed G‐CSF is released into the local and systemic microenvironment. IL‐10 was observed, and lower concentrations of GM‐CSF, IL‐1β and IFN‐γ (Figure 4b and c), which can be further increased by treatment with recombinant G‐CSF (Supplementary figure 7a), consistent with similar populations of CD15^+^CD14^+^ cells, were also observed. The culture of healthy donor granulocytes with sorted decidual cells from placentas led to an upregulation of CD14 on CD15^+^ granulocytes, which was reduced by the addition of G‐CSF blocking antibody (Figure 4d and e). G‐CSF expression was confirmed within the decidua by immunohistochemistry of a placental tissue microarray (Figure 4f). G‐CSF can similarly be found within the tumor microenvironment of common cancer subtypes (Supplementary figure 7b and c). Therefore, G‐CSF released from placental decidual cells or tumor tissue can be one of the causative factors that modulates peripheral blood granulocytes to a CD14^+^CD15^+^ MDSC phenotype, which suppress T‐cell proliferation via ROS production.

The identification of CD15^+^ cells that co‐express CD14 has been documented before, but their functional phenotype and the physiological mechanisms leading to their expansion have not been reported. CD15^+^CD14^+^ cells have been described in severe rheumatoid arthritis where they were considered ‘abnormal myeloid cells’, and *in vitro* could be generated from induced pluripotent stem cells derived from such patients.^23^ In relation to pregnancy, CD14^+^CD15^+^ cells have been observed in foetal cord blood although they were not defined mechanistically.^24^ CD14 itself is classically a receptor for bacterial lipopolysaccharide (LPS), which in conjunction with Toll‐like receptor 4 (TLR4) leads to an intracellular inflammatory signalling cascade.^25^ It also has been reported to bind oxidised phospholipids.^26^ The biological rationale for CD14 upregulation on the subpopulation of immunosuppressive granulocytes identified in this study warrants further research.

Our findings have potential translational consequences, notably in patients with cancer receiving immunotherapies. In such situations, the use of G‐CSF may have a negative effect by rapidly inducing immunosuppressive CD15^+^CD14^+^ cells from peripheral blood granulocytes, which restrict T‐cell anticancer activity. G‐CSF can also mobilise CD34^+^ regulatory monocytes that suppress T‐cell proliferation, and G‐CSF production by tumors can be a negative prognostic factor.^27^ We observed that the dose of G‐CSF given in patients with cancer for stem cell mobilisation is approximately fivefold higher than those given to pregnant women, and may account for the failure to significantly increase double‐positive cells in the miscarriage clinical trial. The retrospective analysis of patient cohorts paints a mixed picture. Treatment of leukaemic patients with G‐CSF post‐mismatched haematopoietic stem cell transplant led to a long‐lasting abnormal myeloid cell antigen presentation and an impaired T‐cell proliferation and response, although controversy remains on its effects in unmanipulated haploidentical allografts based on G‐CSF and ATG regimens.^28^ However, in a cohort of patients treated with anti‐CD19 CAR‐T cells for relapsed/refractory diffuse large B‐cell lymphoma, prophylactic G‐CSF had no negative impact on outcome or CAR‐T cell expansion.^29^ Our findings suggest clinicians should be mindful of the use and scheduling of cytokines alongside immunotherapy. To date, few studies have systematically modelled preclinically or clinically when G‐CSF is best placed alongside immune therapies.^30^ Strategies could include the restriction of G‐CSF to short periods of time post‐cytotoxic chemotherapy to reduce the risk of bacterial infections, before omitting G‐CSF during periods of T‐cell‐activating drugs or adoptive transfer of engineered T cells. Consideration as to whether a cytokine such as G‐CSF will not only expand granulocyte numbers but also negatively modulate T‐cell responses and potential outcomes of immunotherapy should be made in the future clinical trial design.

## Methods

### Sample collection

Heparinised peripheral blood was collected from stage III/IV solid cancer patients with pancreatic cancer (*n* = 10), colon cancer (*n* = 10), breast cancer (*n* = 10), melanoma (*n* = 10), prostate cancer (*n* = 5), head and neck cancer (*n* = 5) and lung cancer (*n* = 5) at diagnosis prior to treatment (*n* = 55). Additional blood was collected from patients with cancer (stage III/IV) undergoing mobilisation of peripheral blood stem cells for harvesting, with 10 µg kg^−1^ filgrastim once a day by subcutaneous injection. Blood was collected 7 days after the start of filgrastim.

Heparinised, peripheral blood was also collected from pregnant women with a history of recurrent miscarriage in conjunction with the RESPONSE trial (*n* = 40).^16^ RESPONSE was a randomised, double‐blind, placebo‐controlled clinical trial for women with a history of unexplained recurrent pregnancy loss conducted in the United Kingdom. Women were randomised to receive placebo or recombinant G‐CSF (130 µg subcutaneously daily) from three to five weeks of gestation for up to nine weeks. The primary outcome was clinical pregnancy at 20 weeks of gestation demonstrated on an ultrasound scan. Secondary outcomes included live birth, miscarriages, adverse events, stillbirth, neonatal birth weight and changes in laboratory parameters.

In accordance with the declaration of Helsinki, patient samples were obtained after written informed consent prior to inclusion in the study (Regional Ethics Committee approval numbers 10/H0501/39 and 14/NW/0130). The University HBRC is licensed by the Human Tissue Authority to collect process and store project‐independent human samples for biomedical research. Samples collected by HBRC are released under Research Tissue Bank ethics approval by the North West Research Ethics Committee, Haydock Park (Ref 15/NW/0079). Blood samples from female, and age‐ and sex‐matched healthy donors were obtained at the University of Birmingham, UK. Healthy leucocyte cones were provided by the NHSBT Blood Bank (Birmingham, UK). The whole blood was lysed using Red Cell Lysis Buffer (Qiagen, Hilden, Germany). Where indicated, peripheral blood was separated using a Lymphoprep density gradient. Human and murine placental tissue samples were digested using type II collagenase (Worthington, Lakewood, USA) for 3 h at 37°C.

### Flow cytometric analysis

Cell populations were identified using anti‐CD15, anti‐CD14, anti‐CD11b, anti‐CD33, anti‐CD62L, anti‐CD10, anti‐LOX1 and anti‐CD16 (eBioscience, San Diego, USA), and anti‐HLA‐DR (BD Bioscience, Oxford, UK) antibodies. Intracellular staining was performed for NOX2, ARG1, IL‐1β, IL‐6, IL‐4, IL‐13 and IL‐10 (eBioscience), and iNOS (Santa Cruz, Dallas, USA) according to the manufacturer’s instructions. T‐cell populations were identified using anti‐CD3, anti‐CD4 and anti‐CD8 antibodies (BD Bioscience, Oxford, UK). Antibody staining was performed on ice for 20 min, and propidium iodide was added to assess cell viability. The immunophenotype was assessed by flow cytometry using a CyAn ADP Analyzer (DakoCytomation, Glostrup, Denmark) in conjunction with the CellQuest software (BD, San Jose, USA). Data were analysed using the FlowJo 8.8 software (Tree Star Inc., Ashland, USA). Where indicated, sorted cells were stained with 2′, 7′‐dichlorofluorescein diacetate using a DCFDA cellular ROS detection assay kit (Abcam, Cambridge, UK) for 30 min at 37°C.

### CD15^+^ cell isolation

CD15^+^ granulocytes were purified from the peripheral blood using a Lymphoprep gradient (STEMCELL Technologies, Saint‐Egreve, France). Leucocytes pelleted as a layer over red blood cells were collected and then purified using anti‐CD15‐coated beads (Miltenyi, Bergisch Gladbach, Germany) according to the manufacturer’s instructions. Where indicated, these cells, or CD15^+^ cells from *in vitro* cultures, were isolated by subsequent sorting with anti‐CD14‐coated beads (Miltenyi) according to the manufacturer’s instructions, followed by flow cytometric confirmation of purity.

### Granulocyte polarisation

To generate placental conditioned media (DCS), decidua were plated (1.5 × 10^6^ cells) and cultured for 48 h. The conditioned media were removed and filtered prior to use. Following Lymphoprep isolation, high‐density granulocytes were enriched by CD15 magnetic bead isolation as above, and healthy donor granulocytes were plated in R10% in 24‐well plates, at concentrations of 1 × 10^6^ per well. DCS was added as 25% of the total volume as indicated. Granulocytes were harvested following 24 h of culture, and washed twice prior to use in suppression assays. Granulocyte viability was confirmed to be > 90% in all cases, by flow cytometry, before further experimentation. Unless otherwise indicated, granulocytes or bone marrow from healthy donors (Lonza, Basel, Switzerland) was polarised using recombinant G‐CSF 1 ng mL^−1^ (PeproTech, UK) for 48 h. Anti‐G‐CSF antibody was added at a concentration of 10 µg mL^−1^.

### T‐cell proliferation assay

T cells (2 × 10^5^) were stimulated with anti‐CD3 (3 µg mL^−1^, BD Bioscience) and anti‐CD2 (2 µg mL^−1^, BD Bioscience) at 37°C and 5% CO2 for 4 days in 96‐well flat‐bottom plates, and then, 1 μCi per well of 3H‐thymidine (PerkinElmer Life Sciences, Beaconsfield, UK) was added for 12–16 h. ^3^H‐thymidine incorporation was measured using a Wallac Microbeta Jet 1450 reader (PerkinElmer, High Wycombe, UK). The suppressive ability of CD15^+^CD14^+^ cells from patients was assessed by co‐culturing purified cells together with allogeneic CD3^+^ T cells, sorted by MACS (Miltenyi). Data are expressed as a percentage of T‐cell proliferation driven by anti‐CD3/CD28 in the presence of CD15^+^CD14^+^ cells compared with T‐cell proliferation in the absence of CD15^+^CD14^+^ cells (100%). Where indicated, 10 mmol L^−1^ (N‐acetylcysteine, iNAC; Sigma‐Aldrich, St. Louis, USA) was added to cell cultures.

### RNA extraction and RT‐qPCR

RNA was extracted from 2 × 10^6^ freshly isolated G‐MDSCs. RNA was extracted with RNeasy columns (Qiagen, Germantown, USA) and quantified with a NanoDrop Spectrophotometer (Thermo Scientific, Waltham, USA). cDNA was synthesised from 200 ng RNA using the High‐Capacity cDNA Archive Kit. Real‐time quantitative PCR was performed in duplicate using the Applied Biosystems 7500 Fast Real‐Time PCR System (Thermo Scientific), according to the manufacturer’s instructions. SYBR Green PCR Master Mix was from Thermo Scientific, and the primers were from Eurofins (Trieste, Italy). Analysis of gene expression was calculated according to the 2^−^Ct^ method and plotted as arbitrary units of mRNA relative to GAPDH. Primer sequences are shown in Supplementary table 1.

### Murine samples

Blood, splenocytes and placenta were harvested from C57BL/6 mice, and procedures were carried out in accordance with UK Home Office Guidelines.

### ELISA

Plasma was collected from pregnant women after sedimentation. Cytokines from plasma and culture supernatants were quantified with ELISA kits according to the manufacturer's instructions (IL‐10, IL‐13, IL‐6 and ELISA kits were supplied by eBioscience, and G‐CSF ELISA kit was supplied by BioLegend, San Diego, USA). Arginine concentrations were measured using a colorimetric assay according to the manufacturer’s instructions (K7733; Immundiagnostik, Bensheim, Germany).

#### Immunohistochemistry

Placental sections from normal term pregnancies (*n* = 12 cases, 24 cores) and tumor sections (*n* = 4 colon adenocarcinoma + 2 healthy colon tissue, *n* = 4 breast invasive ductal carcinoma + 2 healthy breast tissue, *n* = 4 lung carcinoma + 2 healthy lung tissue, and *n* = 4 prostate adenocarcinoma + 2 healthy prostate tissue) were prepared from US Biomax (Rockville). Additional placental samples (*n* = 8 cases) were obtained from normal first‐trimester placentas obtained after surgical termination of pregnancy (gestational age range 8–13 weeks). Full‐thickness placental biopsies were taken from a central location lying between the basal and chorionic plates. The samples of fresh placental tissue were thoroughly washed in 0.9% phosphate‐buffered saline (PBS). Tissues were preserved for immunocytochemistry, with biopsies being fixed in formalin before embedding in paraffin wax. Ethics approval for tissue collection was obtained from the South Birmingham Ethics Committee.

For the TMA, samples were deparaffinised in Histoclear (National Diagnostics, Atlanta) and ethanol, and rehydrated in 0.3% hydrogen peroxide for 15 min. Antigen retrieval was performed in 10 mM sodium citrate buffer (pH 6.0) for 20 min using a microwave oven. Slides were cooled and washed prior to blocking in 5X Casein (Thermo Scientific) for 30 min at room temperature. Sections were then incubated overnight with primary antibody, rabbit anti‐G‐CSF (Abcam).^31^ Sections were washed, and secondary antibody (Universal ImmPRESS antibody, Vector Laboratories, Burlingame, USA) was added at room temperature for 30 min, followed by further washing and addition of DAB substrate (ImmPACT DAB, Vector Laboratories) for 5 min. After counterstaining with Harris’s haematoxylin (Sigma‐Aldrich), slides were dehydrated using ethanol and Histoclear and mounted using OmniMount (National Diagnostics). Slides were examined and photographed using a Nikon Eclipse 400 microscope.

For relevant first‐trimester sections, both single NOX2 (abcam ab80897) and double NOX2/CD15 (Dako, Carb‐3) IHC stainings were performed on the Leica Bond Max autostainer. Slides were dewaxed, and the antigen was retrieved in ER1 (low pH) solution for 20 min. The Bond Polymer Refine Detection Kit was used to detect NOX2 single‐plex staining (Protocol F), whilst the Bond Polymer Refine Detection Kit (Protocol F for double staining) combined with the Bond Refine Red Detection Kit (Protocol J) was used to detect NOX2 and CD15 for dual staining.

#### Statistical analysis

We used *t*‐tests to determine the statistical significance of the difference in paired observations between groups (GraphPad Prism, San Diego, USA). All *P*‐values are two‐tailed, and *P*‐values < 0.05 were considered to represent statistically significant events.

## Conflict of interest

The authors declare no conflict of interest.

## Author contribution

**Ebtehag Maneta:** Data curation; Formal analysis; Investigation; Methodology; Writing – review & editing. **Livingstone Fultang:** Conceptualization; Data curation; Formal analysis; Investigation; Methodology; Writing – review & editing. **Jemma Taylor:** Data curation; Formal analysis; Investigation; Methodology; Writing – review & editing. **Matthew Pugh:** Formal analysis; Investigation; Methodology; Writing – original draft. **William Jenkinson:** Data curation; Investigation; Methodology; Writing – review & editing. **Arri Coomarasamy:** Data curation; Formal analysis; Investigation; Methodology; Writing – review & editing. **Mark Kilby:** Data curation; Formal analysis; Investigation; Methodology; Writing – review & editing. **David Lissauer:** Formal analysis; Funding acquisition; Investigation; Methodology; Writing – review & editing.

## Supporting information

Supplementary figures 1–7

## References

[1] Grover A , Sanseviero E , Timosenko E , Gabrilovich DI . Myeloid‐derived suppressor cells: a propitious road to clinic. Cancer Discov 2021; 11: 2693–2706. 34635571 10.1158/2159-8290.CD-21-0764

[2] Bronte V , Brandau S , Chen SH *et al*. Recommendations for myeloid‐derived suppressor cell nomenclature and characterization standards. Nat Commun 2016; 7: 12150. 27381735 10.1038/ncomms12150 PMC4935811

[3] Srivastava MK , Sinha P , Clements VK , Rodriguez P , Ostrand‐Rosenberg S . Myeloid‐derived suppressor cells inhibit T‐cell activation by depleting cystine and cysteine. Cancer Res 2010; 70: 68–77. 20028852 10.1158/0008-5472.CAN-09-2587 PMC2805057

[4] Rodriguez PC , Ernstoff MS , Hernandez C *et al*. Arginase I‐producing myeloid‐derived suppressor cells in renal cell carcinoma are a subpopulation of activated granulocytes. Cancer Res 2009; 69: 1553–1560. 19201693 10.1158/0008-5472.CAN-08-1921 PMC2900845

[5] Khanna S , Graef S , Mussai F *et al*. Tumour‐derived GM‐CSF promotes granulocyte immunosuppression in mesothelioma patients. Clin Cancer Res 2018; 24: 2859–2872. 29602801 10.1158/1078-0432.CCR-17-3757 PMC6601632

[6] Gneo L , Rizkalla N , Hejmadi R , Mussai F , de Santo C , Middleton G . TGF‐β orchestrates the phenotype and function of monocytic myeloid‐derived suppressor cells in colorectal cancer. Cancer Immunol Immunother e‐pub ahead of print 2 November 2021; 10.1007/s00262-021-03081-5. PMC9188538 34727230

[7] He YM , Li X , Perego M *et al*. Transitory presence of myeloid‐derived suppressor cells in neonates is critical for control of inflammation. Nat Med 2018; 24: 224–231. 29334374 10.1038/nm.4467 PMC5803434

[8] Köstlin N , Kugel H , Rieber N *et al*. Granulocytic myeloid‐derived suppressor cells expand in cord blood and human pregnancy and modulate T cell responses. Eur J Immunol 2014; 44: 2582–2591. 24894988 10.1002/eji.201344200

[9] Pan T , Liu Y , Zhong LM *et al*. Myeloid‐derived suppressor cells are essential for maintaining feto‐maternal immunotolerance via STAT3 signaling in mice. J Leukoc Biol 2016; 100: 499–511. 27203698 10.1189/jlb.1A1015-481RR

[10] Nair RR , Sinha P , Khanna A , Singh K . Reduced myeloid‐derived suppressor cells in the blood and endometrium is associated with early miscarriage. Am J Reprod Immunol 2015; 73: 479–486. 25496212 10.1111/aji.12351

[11] Holmes FA , O'Shaughnessy JA , Vukelja S *et al*. Blinded, randomized, multicenter study to evaluate single administration pegfilgrastim once per cycle versus daily filgrastim as an adjunct to chemotherapy in patients with high‐risk stage II or stage III/IV breast cancer. J Clin Oncol 2002; 20: 727–731. 11821454 10.1200/JCO.2002.20.3.727

[12] Morales‐Molina A , Gambera S , Leo A , Garcia‐Castro J . Combination immunotherapy using G‐CSF and oncolytic virotherapy reduces tumor growth in osteosarcoma. J Immunother Cancer 2021; 9: e001703. 33737338 10.1136/jitc-2020-001703 PMC7978281

[13] Mody R , Yu AL , Naranjo A *et al*. Irinotecan, Temozolomide, and Dinutuximab with GM‐CSF in children with refractory or relapsed neuroblastoma: a report from the children's oncology group. J Clin Oncol 2020; 38: 2160–2169. 32343642 10.1200/JCO.20.00203 PMC7325366

[14] Cheng LL , Guan WJ , Duan CY *et al*. Effect of recombinant human granulocyte colony‐stimulating factor for patients with coronavirus disease 2019 (COVID‐19) and lymphopenia: a randomized clinical trial. JAMA Intern Med 2021; 181: 71–78. 32910179 10.1001/jamainternmed.2020.5503 PMC7489414

[15] Lv M , Zhao XS *et al*. Monocytic and promyelocytic myeloid‐derived suppressor cells may contribute to G‐CSF‐induced immune tolerance in haplo‐identical allogeneic hematopoietic stem cell transplantation. Am J Hematol 2015; 90: E9–E16. 25303038 10.1002/ajh.23865

[16] Eapen A , Joing M , Kwon P *et al*. Recombinant human granulocyte‐ colony stimulating factor in women with unexplained recurrent pregnancy losses: a randomized clinical trial. Hum Reprod 2019; 34: 424–432. 30776296 10.1093/humrep/dey393 PMC6389865

[17] Ladenstein R , Potschger U , Valteau‐Couanet D *et al*. Interleukin 2 with anti‐GD2 antibody ch14.18/CHO (dinutuximab beta) in patients with high‐risk neuroblastoma (HR‐NBL1/SIOPEN): a multicentre, randomised, phase 3 trial. Lancet Oncol 2018; 19: 1617–1629. 30442501 10.1016/S1470-2045(18)30578-3

[18] Glodde N , Bald T , van den Boorn‐Konijnenberg D *et al*. Reactive neutrophil responses dependent on the receptor tyrosine kinase c‐MET limit cancer immunotherapy. Immunity 2017; 47: 789–802. 29045907 10.1016/j.immuni.2017.09.012

[19] Whittle SB , Smith V , Silverstein A *et al*. Is high‐risk neuroblastoma induction chemotherapy possible without G‐CSF? A pilot study of safety and treatment delays in the absence of primary prophylactic hematopoietic growth factors. Pediatr Blood Cancer 2020; 67: e28417. 32729196 10.1002/pbc.28417 PMC7722106

[20] Zhang H , Nguyen‐Jackson H , Panopoulos AD , Li HS , Murray PJ , Watowich SS . STAT3 controls myeloid progenitor growth during emergency granulopoiesis. Blood 2010; 116: 2462–2471. 20581311 10.1182/blood-2009-12-259630 PMC2953883

[21] Lissauer D , Goodyear O , Khanum R , Moss PA , Kilby MD . Profile of maternal CD4 T‐cell effector function during normal pregnancy and in women with a history of recurrent miscarriage. Clin Sci (Lond) 2014; 126: 347–354. 23962040 10.1042/CS20130247

[22] Powell RM , Lissauer D , Tamblyn J *et al*. Decidual T cells exhibit a highly differentiated phenotype and demonstrate potential fetal specificity and a strong transcriptional response to IFN. J Immunol 2017; 199: 3406–3417. 28986438 10.4049/jimmunol.1700114 PMC5679367

[23] Nishimoto N , Murakami M , Ito MN , Saito M , Niwa A , Nakahata T . AB0049 appearance of CD14^+^CD15^+^ population during the differentiation from RA‐IPS cells into monocytes. Ann Rheum Dis 2014; 73(Suppl 2): 820.

[24] Karamitros D , Stoilova B , Aboukhalil Z *et al*. Single‐cell analysis reveals the continuum of human lympho‐myeloid progenitor cells. Nat Immunol 2018; 19: 85–97. 29167569 10.1038/s41590-017-0001-2 PMC5884424

[25] Wright SD , Ramos RA , Tobias PS , Ulevitch RJ , Mathison JC . CD14, a receptor for complexes of lipopolysaccharide (LPS) and LPS binding protein. Science 1990; 249: 1431–1433. 1698311 10.1126/science.1698311

[26] Zanoni I , Tan Y , Di Gioia M *et al*. An endogenous caspase‐11 ligand elicits interleukin‐1 release from living dendritic cells. Science 2016; 352: 1232–1236. 27103670 10.1126/science.aaf3036 PMC5111085

[27] D'Aveni M , Rossignol J , Coman T *et al*. G‐CSF mobilizes CD34^+^ regulatory monocytes that inhibit graft‐versus‐host disease. Sci Transl Med 2015; 7: 281ra42. 10.1126/scitranslmed.3010435 25834108

[28] Volpi I , Perruccio K , Tosti A *et al*. Postgrafting administration of granulocyte colony‐stimulating factor impairs functional immune recovery in recipients of human leukocyte antigen haplotype‐mismatched hematopoietic transplants. Blood 2001; 97: 2514–2521. 11290617 10.1182/blood.v97.8.2514

[29] Galli E , Allain V , Di Blasi R *et al*. G‐CSF does not worsen toxicities and efficacy of CAR‐T cells in refractory/relapsed B‐cell lymphoma. Bone Marrow Transplant 2020; 55: 2347–2349. 32719501 10.1038/s41409-020-01006-x

[30] Salem ML , Nassef M , Abdel Salam SG *et al*. Effect of administration timing of postchemotherapy granulocyte colony‐stimulating factor on host‐immune cell recovery and CD8^+^ T‐cell response. J Immunotoxicol 2016; 13: 784–792. 27417188 10.1080/1547691X.2016.1194917 PMC5669798

[31] Zhang Jy WUP , Chen D *et al*. Vitamin D promotes trophoblast cell induced separation of vascular smooth muscle cells in vascular remodeling via induction of G‐CSF. Front Cell Dev Biol 2020; 8: 601043. 33415106 10.3389/fcell.2020.601043 PMC7783206

