# Supplementary material for: G‐CSF induces CD15+ CD14+ cells from granulocytes early in the physiological environment of pregnancy and the cancer immunosuppressive microenvironment
Source: Clin Transl Immunology. 2022 May 17;11(5):e1395. doi: 10.1002/cti2.1395 (PMC9114661; doi:10.1002/cti2.1395)
Supplement: Supplementary file 1 — Supplementary figures 1–7 [file CTI2-11-e1395-s001.pdf]

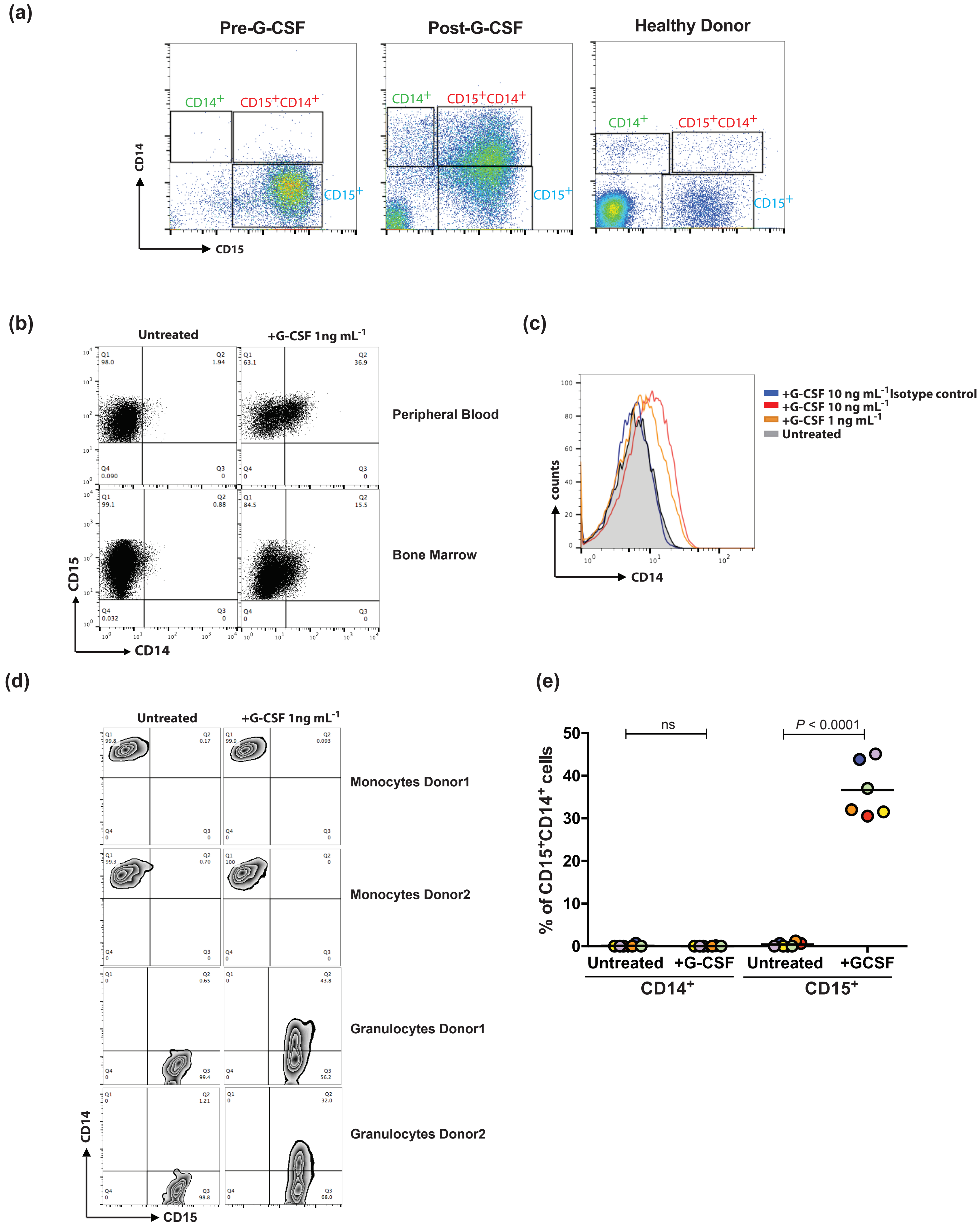

Supplementary Figure 1: Flow characteristics of CD15^{+}CD14^{+} myeloid cells. (a) Representative flow cytometry gating illustrating upregulation of CD14 expression on CD15^{+} granulocytes in the blood of a patient treatedwith recombinant G-CSF. Blood from a healthy donor is shown as a control (b) Representative flow cytometry gating illustrating upregulation of CD14 expression on CD15+ granulocytes from the peripheral blood or bone marrow precursors exposed to G-CSF (c) Expression of CD14 on CD15^{+} granulocytes treated with G-CSF at 1 and 10ng mL^{-1} concentrations for 48hours. In addition CD15^{+} granulocytes treated with 10 ng mL^{-1} G-CSF were stained with mouse IgG1 (isotype control) (d) Representative flow cytometry illustrating the upregulation of CD14 expression on CD15^{+} granulocytes from healthy donors exposed to G-CSF for 48hours. No effect on CD14 or CD15 expression is seen on monocytes (e) pooled analysis of paired samples (n=6).

Supplementary Figure 1

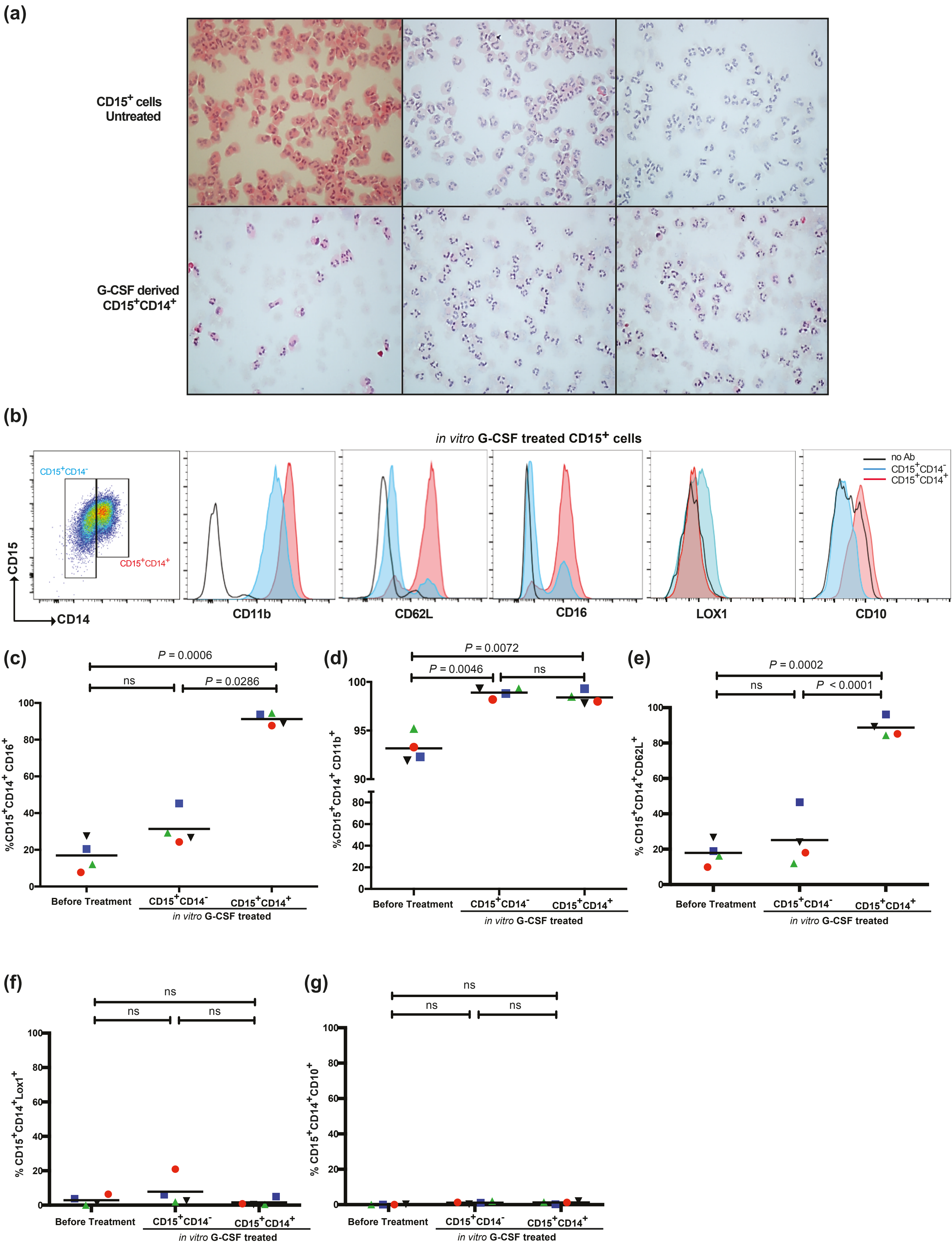

Supplementary Figure 2: Phenotype of $CD15^{+}CD14^{+}$ myeloid cells (a) Haematoxylin and eosin staining of $CD15^{+}CD14^{+}$ cells demonstrating multi-lobed granulocytes, from healthy donor granulocytes treated with G-CSF *in vitro* for 48hours (b) Representative flow cytometry gating illustrating expression of CD11b, CD62L and CD16 on myeloid populations. (c) Pooled analysis of n=4 paired samples
(d) Flow cytometry analysis of granulocytes from n=2 healthy donors showing ROS production (DCFDA staining) on $CD15^{+}CD14^{+}$ cells following G-CSF treatment for 48hours.

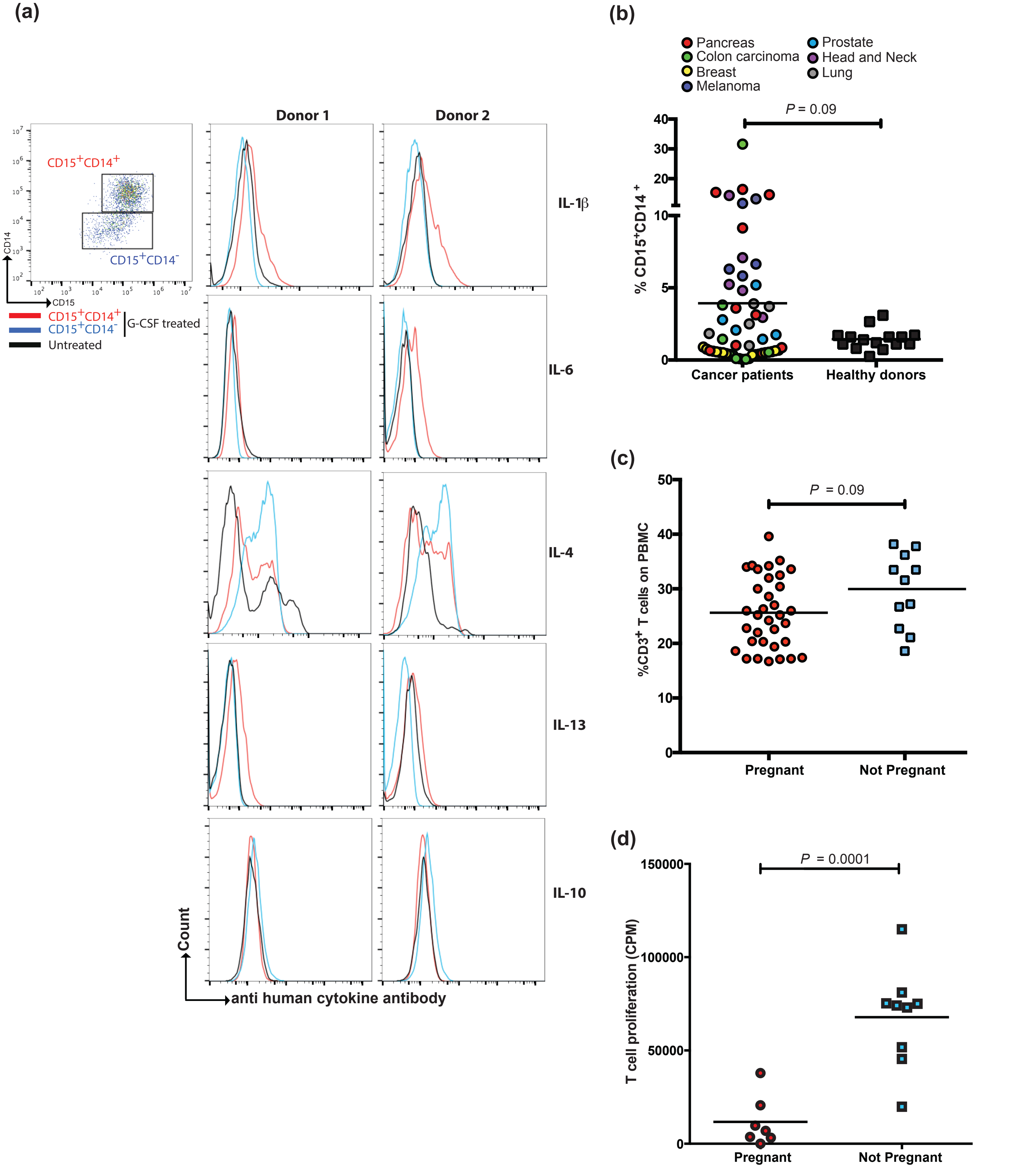

Supplementary Figure 3: Phenotype of CD15+CD14+ myeloid cells (a) Flow cytometry analysis illustrating intracellular expression of cytokines in CD15+CD14+ cells and CD15+CD14- cells following in vitro G-CSF treatment of cells from healthy donors. Untreated CD15+ cells were used as control (b) Frequency of CD15+CD14+ cells in the blood of cancer patients at diagnosis (c) Frequency of CD3+ T cells in the PBMCs of pregnant women at week 6 (d) Capacity of T cells sorted from the blood of pregnant and non-pregnant women to proliferate when cultured in the presence of anti-CD3/anti- CD28 antibodies for 96 hours. Proliferation measured by ^{3}H-thymidine incorporation.

(a)

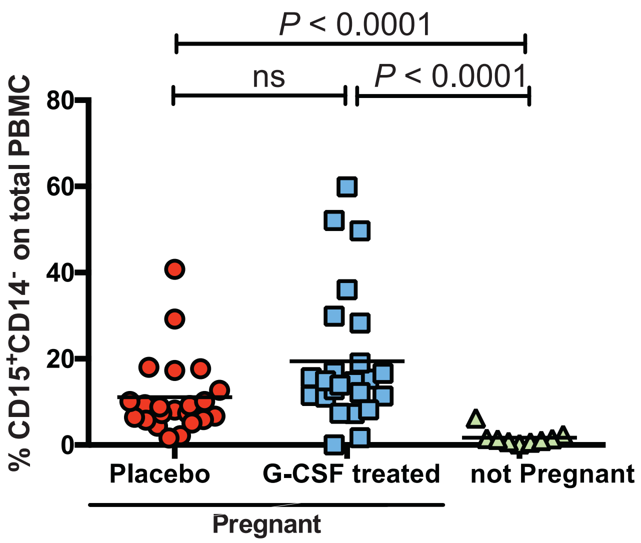

(b)

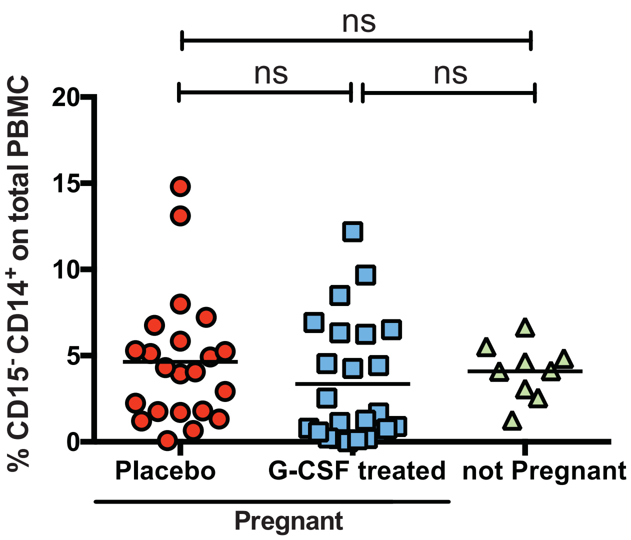

(c)

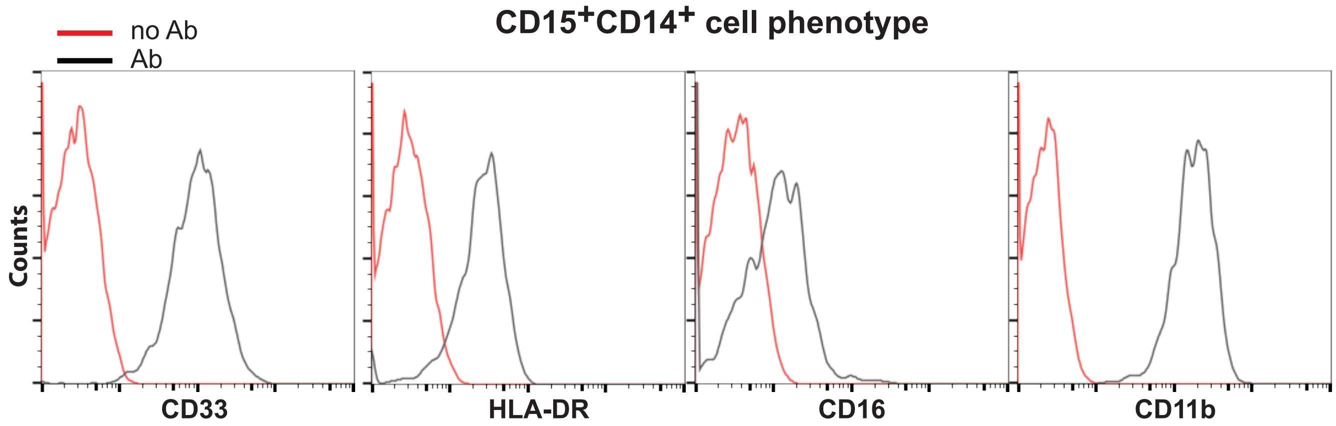

Supplementary Figure 4: Frequency of myeloid cells in pregnant women treated with G-CSF (a) Frequency of $CD15^{+}CD14^{-}$ cells and (b) $CD15^{+}CD14^{+}$ cells at week 6 in the blood of pregnant women randomized to receive recombinant G-CSF (n=24) or placebo (n=24) and non-pregnant controls (n=9) (c) Representative flow cytometry gating illustrating expression of CD33, CD11b, HLA-DR and CD16 on $CD15^{+}CD14^{+}$ cells from the blood of pregnant women.

(a)

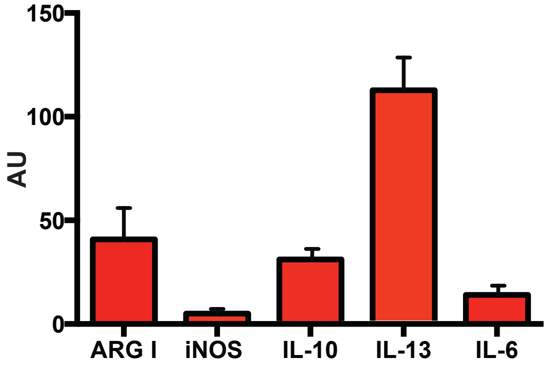

(b)

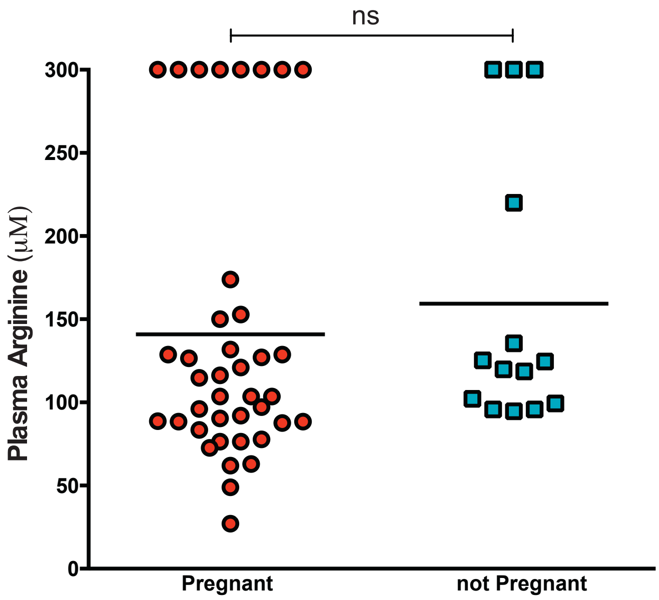

(c)

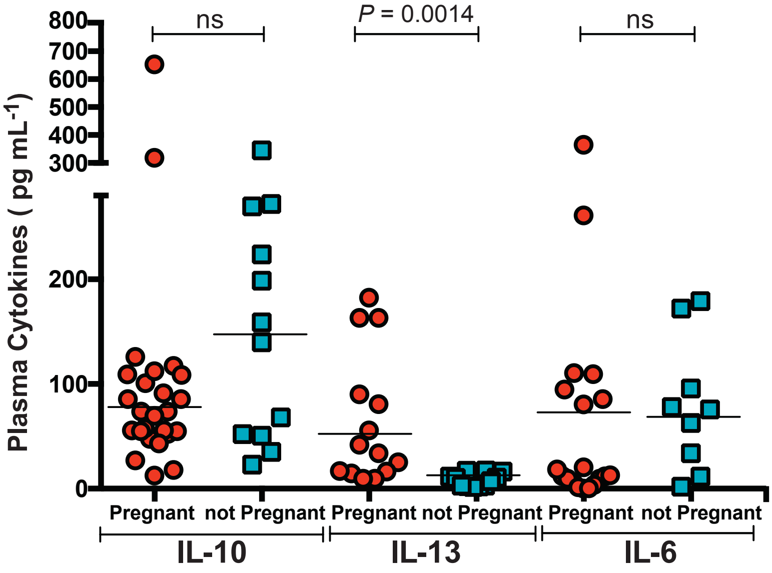

Supplementary Figure 5: Characterisation of potential immunosuppressive mediators (a) qPCR for Arginase I, iNOS, IL-10, IL-13, and IL-6 in sorted $CD15^{+}CD14^{+}$ cells sorted from the blood of pregnant women, as measured by qRT-PCR. Concentrations of (b) arginine and (c) cytokines in the plasma of pregnant women at week 6 or non-pregnant controls as measured by ELISA.

(a)

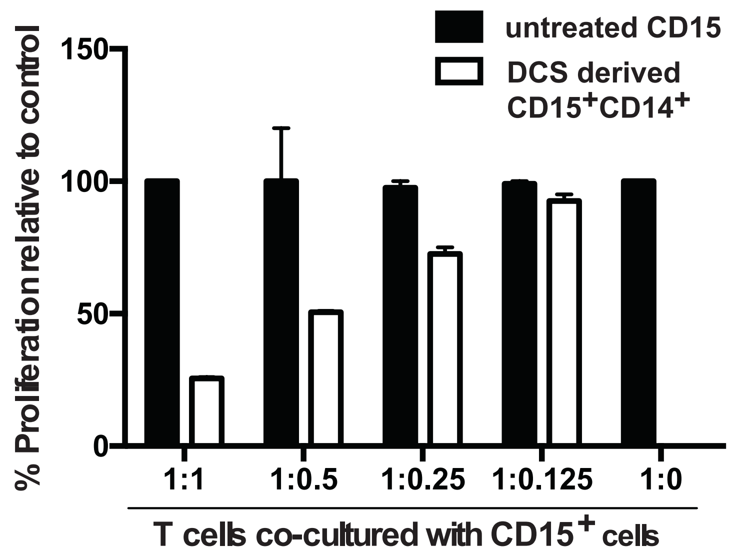

(b)

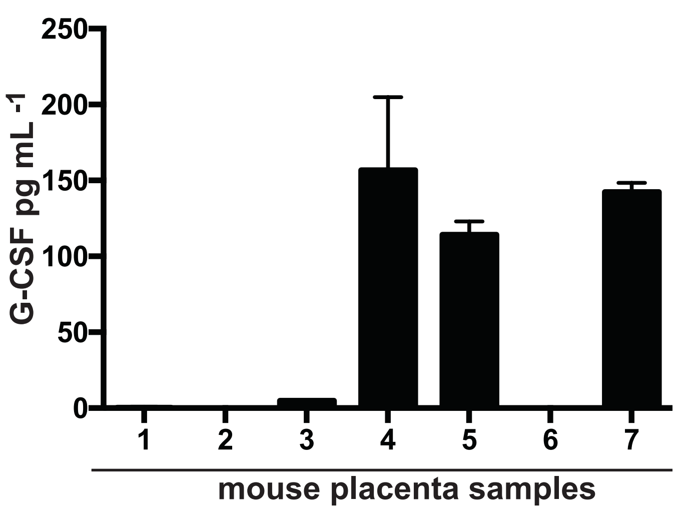

(c)

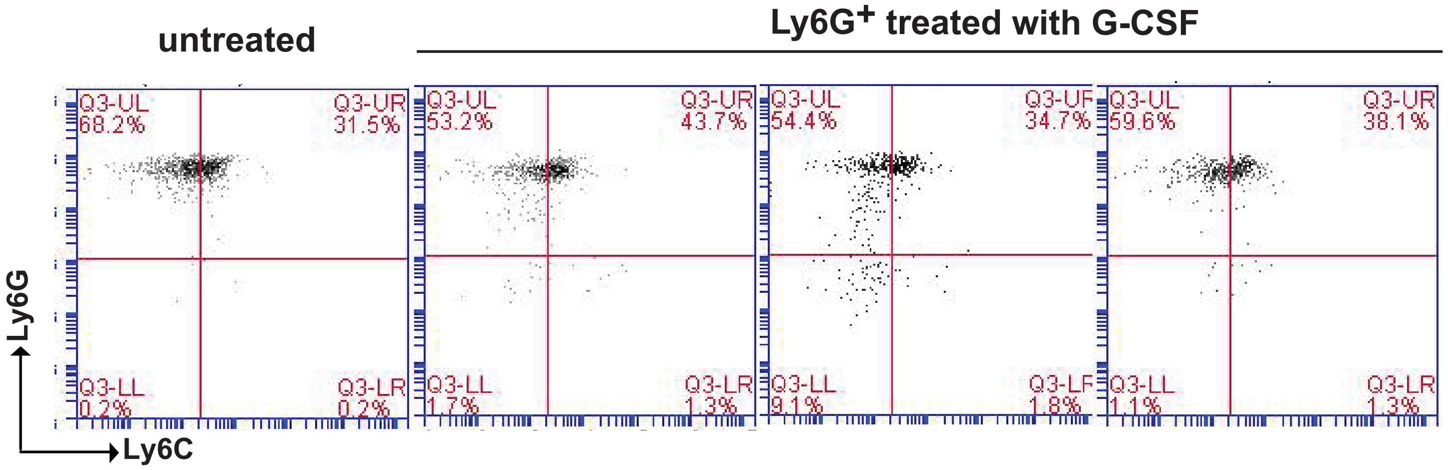

(e)

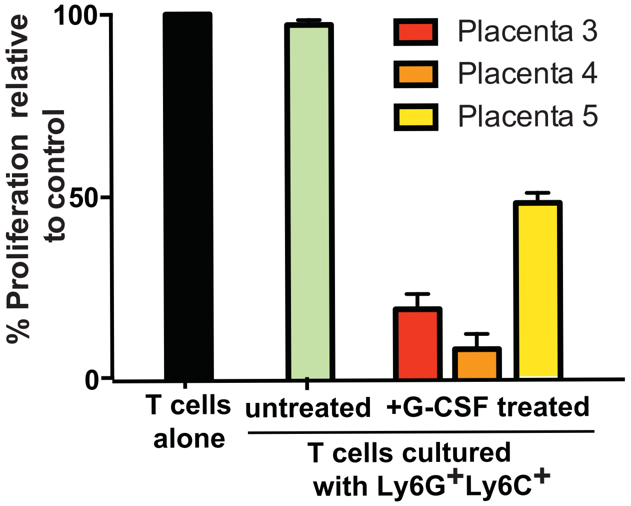

Supplementary Figure 6: $CD15^+CD14^+$ cells within human and murine placental tissues (a) Tissue microarray of human placental tissue (n=12) stained for NOX2 positive myeloid cells (brown) (b) $CD15^+CD14^+$cells, generated by culture of granulocytes in decidua conditioned media, suppress T cell proliferation, stimulated by anti-CD3/CD28 antibodies for 96 hours as measured by ^{3}H-thymidine incorporation (c) ELISA of murine placental cell culture supernatants demonstrating release of G-CSF (n=7) (d) Representative flow cytometry gating illustrating upregulation of Ly6C expression on murine $Ly6G^+$ granulocytes exposed to G-CSF (e) Granulocytes from the spleens of naïve mice conditioned with murine placental supernatants, similarly suppress murine T cell proliferation as measured by ^{3}H- thymidine incorporation (n=3)

(a)

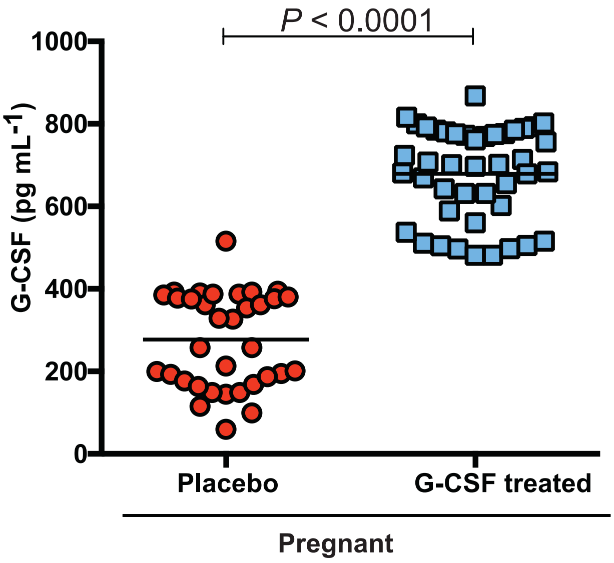

(b)

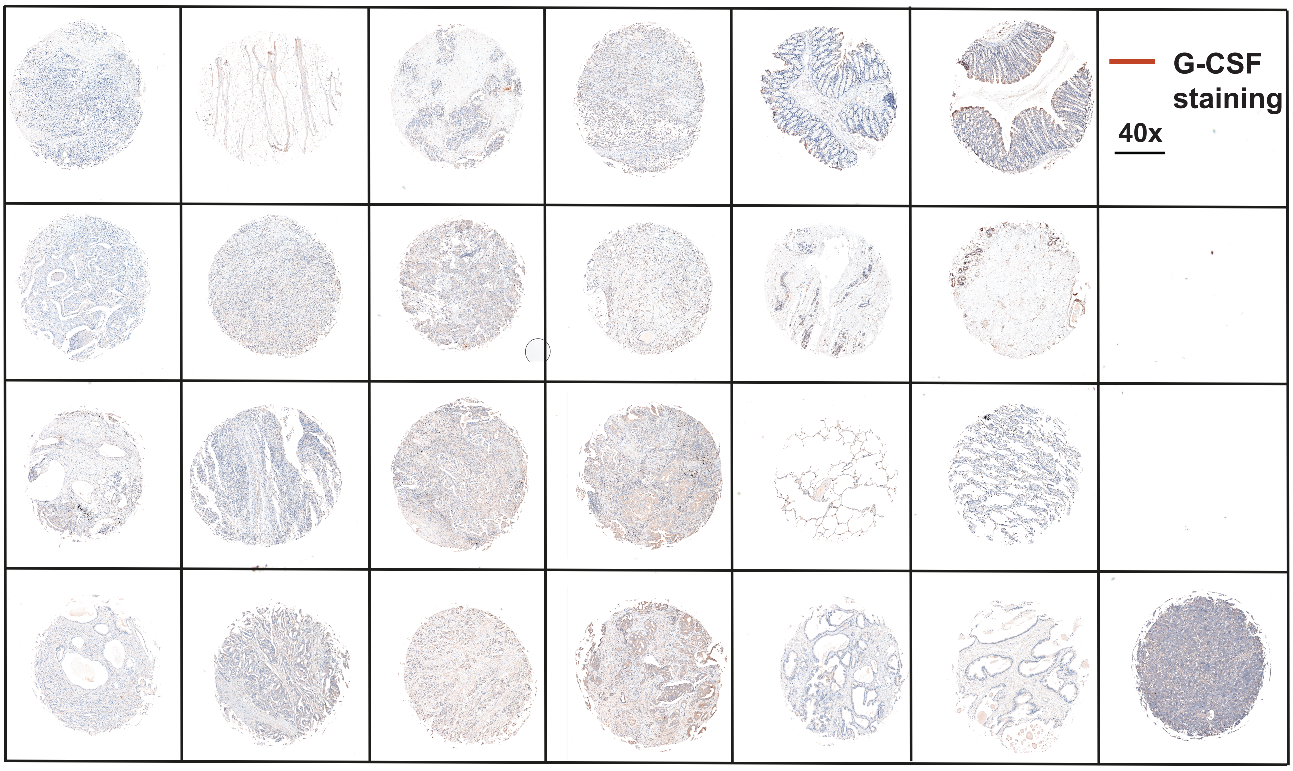

(c)

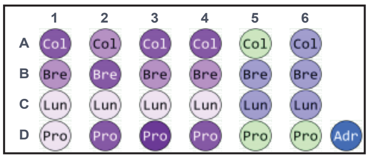

Legend:Bre - Breast, **Col** - Colon, **Lun** - Lung, **Pro** - Prostate

- Malignant tumor
- Malignant tumor (stage I)
- Malignant tumor (stage IB),
- Malignant tumor (stage IIA)
- Malignant tumor (stage IIB)
- Malignant tumor (stage III)
- Malignant tumor (stage IIIB)
- Malignant tumor (stage IIIC)
- Malignant tumor (stage IV)
- NAT
- Normal tissue

Pos.	No.	Age	Sex	Organ/Anatomic Site	Pathology diagnosis	TNM	Grade	Stage	Type
A1	1	81	F	Colon	Mucinous adenocarcinoma	T4N2M0	3	IIIC	Malignant
A2	2	44	F	Colon	Mucinous adenocarcinoma	T3N0M0	3	IIA	Malignant
A3	3	40	M	Colon	Adenocarcinoma	T4N2M0	3	IIIC	Malignant
A4	4	56	M	Colon	Adenocarcinoma	T4N2M0	3	IIIC	Malignant
A5	5	45	M	Colon	Colon tissue	-	-	-	Normal
A6	6	69	M	Colon	Adjacent normal colon tissue	-	-	-	NAT
B1	7	58	F	Breast	Invasive ductal carcinoma	T2N1M0	2	IIB	Malignant
B2	8	50	F	Breast	Invasive ductal carcinoma	T4N1M0	2	IIIB	Malignant
B3	9	42	F	Breast	Invasive ductal carcinoma	T2N0M0	3	IIA	Malignant
B4	10	56	F	Breast	Invasive ductal carcinoma	T2N1M0	3	IIB	Malignant
B5	11	46	F	Breast	Adjacent normal breast tissue (adenosis)	-	-	-	NAT
B6	12	37	F	Breast	Adjacent normal breast tissue	-	-	-	NAT
C1	13	66	M	Lung	Squamous cell carcinoma	T2N0M0	2	IB	Malignant
C2	14	70	M	Lung	Squamous cell carcinoma (sparse)	T2N0M0	3	IB	Malignant
C3	15	71	M	Lung	Adenocarcinoma	T2N0M0	2	IB	Malignant
C4	16	69	F	Lung	Adenocarcinoma	T2N0M0	2	IB	Malignant
C5	17	52	M	Lung	Adjacent normal lung tissue	-	-	-	NAT
C6	18	52	M	Lung	Adjacent normal lung tissue	-	-	-	NAT
D1	19	71	M	Prostate	Adenocarcinoma Gleason 2 (2+2)	T2N0M0	1	I	Malignant
D2	20	73	M	Prostate	Adenocarcinoma Gleason 4 (4+4)	T3N0M0	2	III	Malignant
D3	21	60	M	Prostate	Adenocarcinoma Gleason 3 (3+3)	T3N1M0	2	IV	Malignant
D4	22	64	M	Prostate	Adenocarcinoma Gleason 4 (4+3)	T3N0M0	2	III	Malignant
D5	23	31	M	Prostate	Prostate tissue	-	-	-	Normal
D6	24	35	M	Prostate	Prostate tissue	-	-	-	Normal
-	0	42	M	Adrenal gland	Pheochromocytoma (tissue marker)		-		Malignant

Supplementary Figure 7: G-CSF expression in pregnant women and cancer patients (a) Concentration of G-CSF in the plasma of pregnant women randomized to receive recombinant G-CSF (n=43) or placebo (n=34) as measured by ELISA (b) Tissue microarray of human tumour tissue stained for G-CSF (brown) and associated sample key (c)
